# Public Service by a Selfish Gene: A Domesticated Transposase Antagonizes Polycomb Function

**DOI:** 10.1371/journal.pgen.1006014

**Published:** 2016-06-02

**Authors:** William A. Ricci, Xiaoyu Zhang

**Affiliations:** Department of Plant Biology, University of Georgia, Athens, Georgia, United States of America; University of Utah School of Medicine, UNITED STATES

Eukaryotic genomes are littered with transposable elements (TEs)—“selfish” genetic entities capable of increasing copy numbers through transposition to account for large fractions of the nuclear DNA. To minimize the mutagenic effects of transposition, host organisms have evolved various mechanisms to repress TE-encoded genes (TEGs) both transcriptionally through chromatin modifications and post-transcriptionally through RNA interference. Over time, silenced TEs accumulate genetic mutations, become immobilized, and are eliminated from the genome by recombination or decay into intergenic DNA. But can immobilized TEGs fortuitously acquire cellular functions that are beneficial to the host and become a useful fixture of the genome? In a recent issue of *PLOS Genetics*, Turck, Goodrich, and colleagues describe a clear example of this process, in which a transposase-derived gene functions to antagonize transcriptional repression by the Polycomb group (PcG) genes in *Arabidopsis thaliana* [1].

PcG genes play critically important roles in regulating plant development by targeting thousands of genes for transcriptional repression through trimethylation of lysine 27 on histone H3 (H3K27me3) [2]. In *Arabidopsis*, PcG function requires two classes of protein complexes: Polycomb Repressive Complexes 1 and 2 (PRC1 and PRC2, respectively). The enzymatic complex PRC2 contains one of the three H3K27 trimethyltransferases, MEDEA (MEA), CURLY LEAF (CLF), and SWINGER (SWN), whereas the PRC1 complex includes the H3K27me3-binding protein LIKE HETEROCHROMATIN PROTEIN 1 (LHP1). To better understand the mechanisms that may counteract PcG repression, two independent suppressor screens were performed to identify mutants that could revert the developmental and transcriptional defects of the CLF and LHP1 mutants, respectively [1,3]. One gene named *ANTAGONIST OF LIKE HETEROCHROMATIN PROTEIN 1* (*ALP1*) was isolated from both screens, indicating that it likely functions broadly in antagonizing PcG repression [1,3]. This notion is supported by several lines of evidence: (1) *alp1* suppresses the developmental phenotypes of *clf*; (2) a significant faction of genes overexpressed in *clf* are no longer overexpressed in *alp1 clf*; (3) *ALP1* functions upstream of PcG-repressed genes (e.g., *AGAMOUS*); (4) many PcG-target genes are down-regulated in *alp1* when normal PcG activity is present; (5) *alp1* enhances the defects of several mutants of trithorax group (trxG) genes involved in counteracting PcG repression; and (6) ALP1 physically interacts with PRC2 *in planta*. Taken together, these results strongly indicate that *ALP1* is generally required to antagonize PcG repression at a large number of developmentally important genes [1].

Interestingly, *ALP1* encodes a protein that is highly similar to the transposases (TPases) of the *PIF*/*Harbinger* superfamily of DNA TEs [1,3]. The first active member of the superfamily, *P Instability Factor* (*PIF*), was identified in maize as repeated mutagenic insertions into the anthocyanin regulatory gene *R* and was later found to be similar to the *Harbinger* elements computationally identified from *Arabidopsis* [4–6]. The *PIF*/*Harbinger* superfamily is distantly related to the bacterial *IS5* elements and includes five major groups in eukaryotes: two separate plant-specific groups (*PIF-* and *Pong*-like groups), an animal-specific group, and two fungal groups [7]. Members of the *PIF*/*Harbinger* superfamily share several characteristics: they have short terminal inverted repeats (TIRs), prefer to insert into 3-bp target sites embedded in longer palindromic sequences, and encode two proteins (a Myb-like DNA-binding protein and a TPase) that are both required for transposition [8,9]. *ALP1* shares extensive homology with *PIF*-like TPases. However, there are several important differences: (1) *PIF*- and *Pong*-like elements are present at moderate-to-high copy numbers in plant genomes (from dozens in *Arabidopsis* to ~1,000 in *Brassica oleracea*), whereas *ALP1*-like genes are present at single copy in land plants; (2) the DDE catalytic motif in *PIF*-like TPases is mutated in ALP1 and its homologues in angiosperms (but not in gymnosperms, ferns, bryophtyes, and green algae) and the Myb-like gene and TIRs are missing from *ALP1* flanking sequences; and (3) the majority of *PIF*-like elements are transcriptionally silent and have accumulated missense or nonsense mutations, whereas *ALP1* is broadly expressed in *Arabidopsis* leaves, stems, flowers, and roots, and its coding capacity is well preserved in land plants [1]. Taken together, these results suggest that *ALP1* likely originated from a *PIF*-like TPase gene and acquired an important cellular function in the common ancestor of angiosperms.

In addition to *ALP1*, a number of TPase-derived genes have been described in eukaryotes (for excellent recent reviews, see [10,11]). The majority of such genes were computationally identified based on the set of characteristics that distinguish *ALP1* from *PIF* TPases, including loss of catalytic activity for transposition, high degree of evolutionary conservation, being present at low copy number, and evidence for transcriptional activity. Importantly, several TPase-derived genes have been shown to provide vital functions for the hosts. For example, the SETMAR protein—created from a fusion between a *Mariner* TPase and a SET histone methyltransferase domain—is required for the maintenance of genome integrity in primates [12,13]. In plants, the *Arabidopsis DAYSLEEPER* gene encodes a DNA-binding protein derived from a TPase of the *hAT* superfamily. DAYSLEEPER binds to a *cis*-regulatory motif upstream of multiple genes and the *daysleeper* mutant displays severe and pleiotropic developmental phenotypes [14]. As another example, *FHY3* and *FAR1* are derived from the *MURA* TPase gene encoded by Mutator-like elements (MULEs) [15]. However, both *FHY3* and *FAR1* function as transcription factors that activate gene expression under far-red light.

How ALP1 antagonizes PcG repression and how a TPase acquired such a function remain open questions. Based on the observation that the interactions of PRC1 and ALP1 with PRC2 appeared to be mutually exclusive, Liang et al. proposed that ALP1 may compete with PRC1 for binding to PRC2 and thereby alleviate PcG repression [1]. In this regard, it is interesting to note that, during *PIF* transposition, the TPase is recruited to TEs by interacting with the Myb-domain protein, which in turn binds specific DNA sequences at TE ends [9]. It is therefore possible that a catalytically inactive mutant TPase with altered preference for protein–protein interactions might have fortuitously acquired PRC2-binding activity. It is also possible that, considering the role of PcG as a “backup system” to repress TE activity (behind DNA methylation) [16], the interaction of a TPase with PRC2 may have originally evolved as an anti-repression mechanism by the TE. Future work should address these questions, for example, by determining whether the same domain is involved in ALP1–PRC2 and TPase–Myb-protein interactions. With the rapid advances of genomic resources and reverse-genetic tools, *ALP1* should serve as harbinger of the identification and functional characterization of many more selfish genes that have evolved to serve their hosts.
